# Asymptomatic choroid plexus carcinoma in an infant: Report of one case

**DOI:** 10.1016/j.amsu.2022.103755

**Published:** 2022-05-11

**Authors:** Mohammed Moutaz Alshaghel, Wafik Mayo, Najeeb Sakkal, Kutaiba Alali, Mahmoud Wereeki

**Affiliations:** a Faculty of Medicine, University of Aleppo, Aleppo, Syria; b CME Office, Faculty of Medicine, University of Aleppo, Aleppo, Syria; c Department of Neurosurgery, Aleppo University Hospital, Aleppo University, Aleppo, Syria; d Mashabek, Aleppo, Syria

**Keywords:** Carcinoma, Choroid, Neurosurgery, Plexus, Ventricle

## Abstract

**Introduction:**

Choroid Plexus Carcinomas (CPC) are rare malignant brain neoplasms of choroid plexus epithelium, with a tendency to occur in infants and children, especially those who are under two years of age. The Main symptoms of CPC include nausea, vomiting, headache, irritability, blurred vision, and seizures. Few studies discuss the therapeutic methods to treat this tumor. However, most of these studies confirmed the poor prognosis of it.

**Case presentation:**

A two-year-old girl presented with a headache due to head trauma, normal consciousness, GCS 15/15, and without intracranial hypertension symptoms. Computed Tomography (CT) has shown a large heterogeneous lesion in the region of the right lateral ventricle. Magnetic resonance imaging (MRI) showed a large poorly-defined mass in the right lateral ventricle with mild dilatation of the ipsilateral lateral ventricle, and midline shift and marked edema surrounding it. In this case, the mass has been discovered by accident. The histological diagnosis was choroid plexus carcinoma (WHO grade 3), curettage of the right lateral ventricle was performed.

**Discussion and conclusion:**

CPC is a serious condition with a poor prognosis. Early diagnosis and appropriate approaches are required in order to reduce mortality and morbidity rates.

## Highlights

• Diagnosis difficulties of Choroid Plexus carcinoma make it an important case.
• Correct diagnosis is often delayed, resulting in a poor prognosis.
• CPC should be present in the diagnosis of atypical brain tumors, especially in children and their atypical symptoms.
• An early and accurate diagnosis of the lesion can improve the prognosis and maintain the patient's quality of life.

## 1 Introduction

Choroid plexus tumors are uncommon intracranial neoplasms, representing approximately 0.4–0.6% of all brain tumors and about 5% of all pediatric tumors. There are two main types, malignant and benign, classiﬁed as choroid plexus carcinoma (CPC) and choroid plexus papilloma (CPP), respectively [1].

CPC represents about 17–30% of all choroid plexus tumors with a tendency to arise in patients younger than two years in comparison with adults [2,3]. CPC is classiﬁed as a highly aggressive malignant neoplasm with Grade III according to the World Health Organization (WHO) [1].

Current treatment strategies range from total gross surgical resection alone to a combination of surgical, radiological, and chemotherapy for both CPC and CPP; however, the medical literature recommends total surgical resection for children with CPC [4,5].

We report a new case of choroid plexus carcinoma diagnosed in a 2-year-old girl in which the clinical outcomes were described.

## 2 Case report

A two-year-old girl presented to the emergency department complaining of headache due to head trauma, with no seizures, no vomiting, and no loss of consciousness. Her physical examination showed increased head size, GCS 15/15, and jerky gait.

**Fig. 1:**
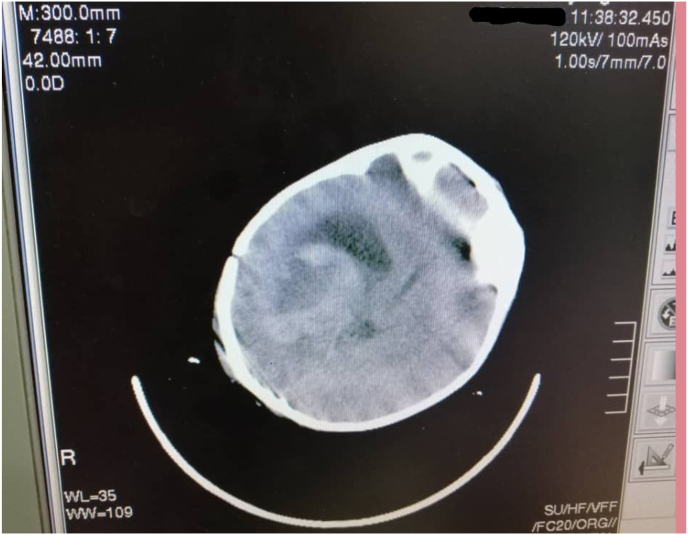
Computed Tomography showing a heterogeneous mass in the right lateral ventricle with marked midline shift.

Computed Tomography (CT) has shown a large heterogeneous lesion with irregular margins, and perilesional edema in the region of the right lateral ventricle, with marked midline shift that compresses the opposite lateral ventricle (Fig. 1).

**Fig. 2:**
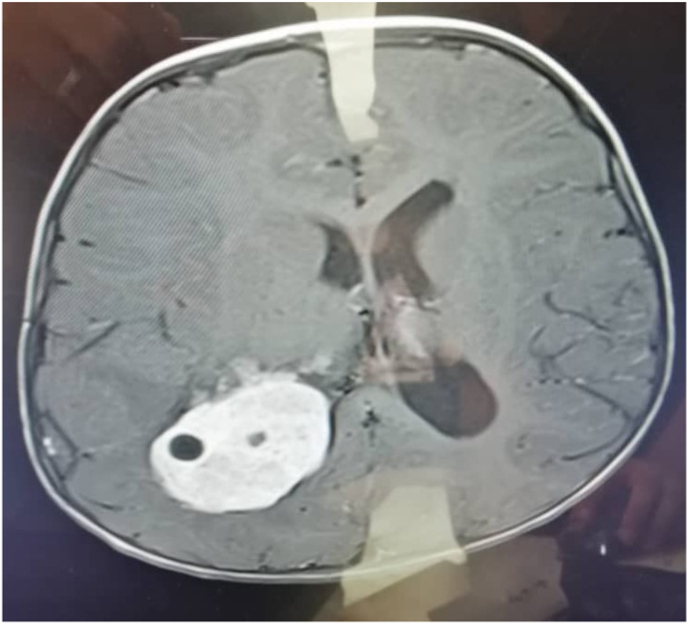
Axial T1 image showing a hypointense lesion.

Magnetic resonance imaging (MRI) showed a large poorly-defined lesion in the right lateral ventricle which appeared to be hypointense on T1 images and hyperintense on T2 images. The mass also showed strong heterogeneous gadolinium enhancement, with mild dilatation of the ipsilateral lateral ventricle, and with midline shift and marked edema surrounding it (Fig. 2). Through a lateral occipital trans sulcal approach, we partially excised a highly vascularized, purple-colored tumor, with solid and soft content from intra- and extra-ventricular. Surgical intervention was applied from neurological residents in the fifth year in the main university hospital of the city.

**Fig. 3:**
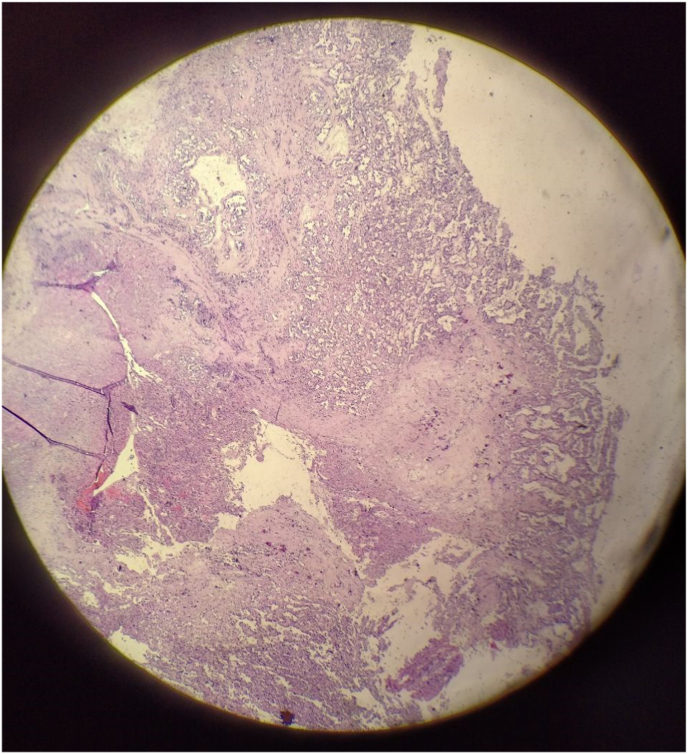
The histological examination shows anaplasia, including nuclear atypia, cellular pleomorphism, increased mitotic activity, focal necrosis, and stromal calcifications. (X20).

The histological examination showed a conspicuous papillary pattern, with tumor cells arranged in dense sheets. Frank features of anaplasia were also presented, including nuclear atypia, cellular pleomorphism, increased mitotic activity, focal necrosis, and stromal calcifications (Fig. 3).

Immunohistochemically, the tumor cells were negative for glial fibrillary acidic protein (GFAP), synaptophysin, and epithelial membrane antigen (EMA). The ki67 was more than 60%.

The histological diagnosis was choroid plexus carcinoma (WHO grade 3), curettage of the right lateral ventricle was performed.

This case report has been reported in line with the SCARE Criteria [6].

## 3 Discussion

Choroid plexus tumors (CPTs) are exceptionally uncommon. They make up less than 4% of all brain tumors in childhood. CPTs are divided into Choroid Plexus Papilloma (CPP) and Choroid plexus carcinoma (CPC) which is rarer and classified as highly invasive tumor according to the classification of the World Health Organization (WHO grade III), accounting for 1% of all CNS tumors [7,8]. The first reports of CPC are believed to date back to 1844 [9].

The tumor arises from the epithelium of the choroid plexuses. The average age of the tumor onset in childhood is in the third year [8]. The lateral ventricles are the most common site of the tumor incidence (50%), followed by the fourth ventricle (40%) and the third ventricle (5–10%); multiple ventricles have been recorded. Some cases have also been recorded in unusual places, such as the angle of the cerebellum. Tumors can be extra ventricular or intraventricular, with a 65% tendency to affect males [[7], [8], [9]].

The etiology of the tumor is not yet clear, but it is believed that a mutation in the TP53 gene which causes Li-Fraumeni syndrome has a strong association with the malignancy incidence. The basis of this claim is that half of the malignant tumor cases are associated with a mutation in this gene, and unfortunately with a worse prognosis [9].

The manifestations and signs of the tumor are similar to any intracranial mass, where intracranial pressure increases and neurological symptoms appear, including headache, vomiting, and nausea. Symptoms and signs can be distinguished in an infant as we note increased head circumference, bulging fontanelles, splayed cranial sutures, and neurological delay [8,9]. What is noteworthy in our case is that the tumor was totally asymptomatic and was discovered by chance through a CT image of the head after a traumatic accident.Several differential diagnoses are consistent with the mentioned symptoms, including choroid plexus papilloma, papillary ependymoma, and metast atic papillary adenocarcinoma [10].The appropriate diagnosis is made through radiographic and histological studies. Radiography, including CT and MRI, demonstrates a large contrast-enhancing mass. It is usually associated with hydrocephalus and edema and shows high density and calcification.

Histological studies revealed increased mitotic figures and nuclear pleomorphism. Moreover, Russell and Rubinstein established specific criteria for an accurate histological diagnosis, including clear invasion of adjacent neural tissue with an ill-defined pattern of growth, loss of papillary architecture (at least in the invaded areas that are associated with mitoses), varying nuclear size, and chromatin content, and cellular pleomorphism.

Immunohistochemically, positive markers of CPC include cytokeratins, vimentin, S100, and transthyretin. Glial fibrillary acidic protein is also positive for CPC, but only in 20% of the cases. CPC is negative for epithelial membrane antigen in general [8,9,11].

Due to the rarity of CPC cases, there is no established protocol approved yet to treat CPC. However, gross total resection is the most accepted treatment, because it improves prognosis, despite the high risk of surgery due to hemorrhage issues and tumor infiltration which results in increased morbidity and mortality chances. About 40–75% of CPC cases applied surgery as a treatment method. The adjuvant therapy is still controversial. In some cases, chemotherapy was applied prior to surgery in order to reduce the tumor's vascularity. Radiation treatment has risks that could not be expected in the pediatric population, as they are the most affected sample by the tumor [[7], [8], [9]].

The prognosis of CPC is poor in general, as the tumor grows rapidly and has an average survival of 2.5–3 years approximately, and recurrence is common and occurs when malignant infiltrates grow rapidly [8,9].

Metastases are apparent at presentation in less than a quarter of cases and are located in areas of tumor infiltration (CSF pathways) [9].

In conclusion, CPCs are rare tumors that emerge in the lateral and fourth ventricles and affect early childhood patients in most cases. Diagnosis is directed by Radiological examinations and confirmed by both histological and immunohistochemical studies. There are no completely approved treatments, but surgery remains the best approach. CPCs have a poor prognosis and recurrence is common.

## 4 Informed consent

As the patient is a child of two years of age, the consent was obtained from the patient's parents.

## Provenance and peer review

Not commissioned, externally peer reviewed.

## Ethical approval

N/A.

## Please state any sources of funding for your research

N/A.

## Author contribution

Kutaiba Alali, Mahmoud Wereekia, and Najeeb Sakkal did the surgery. -Mohammed Moutaz Alshaghel, Wafik Mayo, and Najeeb Sakkal wrote the manuscript.

## Please state any conflicts of interest

Authors declare that there is no conflict of interest.

## Registration of research studies

Name of the registry:

Unique Identifying number or registration ID:

Hyperlink to your specific registration (must be publicly accessible and will be checked):

## Consent

Written informed consent was obtained from the patient for publication of this case report and accompanying images. A copy of the written consent is available for review by the Editor-in-Chief of this journal on request.

## Guarantor

Mahmoud Wereekia

dr.kutaiba90@gmail.coms.
